# The Burnout PRedictiOn Using Wearable aNd ArtIficial IntelligEnce (BROWNIE) study: a decentralized digital health protocol to predict burnout in registered nurses

**DOI:** 10.1186/s12912-024-01711-8

**Published:** 2024-02-13

**Authors:** Angelina R. Wilton, Katharine Sheffield, Quantia Wilkes, Sherry Chesak, Joel Pacyna, Richard Sharp, Paul E. Croarkin, Mohit Chauhan, Liselotte N. Dyrbye, William V. Bobo, Arjun P. Athreya

**Affiliations:** 1 Dept. of Molecular Pharmacology and Experimental Therapeutics, Mayo Clinic, Rochester, MN USA; 2 Division of Nursing Research, Mayo Clinic, Jacksonville, FL USA; 3 Dept. of Nursing, University of Minnesota School of Nursing, Rochester, MN USA; 4 Dept. of Quantitative Health Sciences, Mayo Clinic, Rochester, MN USA; 5 Dept. of Psychiatry and Psychology, Mayo Clinic, 200 First St SW, Rochester, MN 55902 USA; 6 Dept. of Psychiatry and Psychology, Mayo Clinic, 4315 Pablo Oaks Ct, Jacksonville, FL USA; 7 Dept. of Medicine, University of Colorado Anschutz School of Medicine, Aurora, CO USA; 8 Dept. of Medicine, Mayo Clinic Alix School of Medicine, Rochester, MN USA

**Keywords:** Burnout, Wearables, Artificial intelligence, Machine learning

## Abstract

**Background:**

When job demand exceeds job resources, burnout occurs. Burnout in healthcare workers extends beyond negatively affecting their functioning and physical and mental health; it also has been associated with poor medical outcomes for patients. Data-driven technology holds promise for the prediction of occupational burnout before it occurs. Early warning signs of burnout would facilitate preemptive institutional responses for preventing individual, organizational, and public health consequences of occupational burnout. This protocol describes the design and methodology for the decentralized Burnout PRedictiOn Using Wearable aNd ArtIficial IntelligEnce (BROWNIE) Study. This study aims to develop predictive models of occupational burnout and estimate burnout-associated costs using consumer-grade wearable smartwatches and systems-level data.

**Methods:**

A total of 360 registered nurses (RNs) will be recruited in 3 cohorts. These cohorts will serve as training, testing, and validation datasets for developing predictive models. Subjects will consent to one year of participation, including the daily use of a commodity smartwatch that collects heart rate, step count, and sleep data. Subjects will also complete online baseline and quarterly surveys assessing psychological, workplace, and sociodemographic factors. Routine administrative systems-level data on nursing care outcomes will be abstracted weekly.

**Discussion:**

The BROWNIE study was designed to be decentralized and asynchronous to minimize any additional burden on RNs and to ensure that night shift RNs would have equal accessibility to study resources and procedures. The protocol employs novel engagement strategies with participants to maintain compliance and reduce attrition to address the historical challenges of research using wearable devices.

**Trial Registration:**

NCT05481138.

**Supplementary Information:**

The online version contains supplementary material available at 10.1186/s12912-024-01711-8.

## Background

 The World Health Organization characterizes burnout in the 11th Revision of The International Classification of Diseases (ICD-11) as “a syndrome in which chronic workplace stress has not been successfully managed.” The job demands-resources (JD-R) model for burnout and life satisfaction proposes that excessive demands at work lead to a depletion of emotional resources that lead to exhaustion. A lack of workplace resources to combat excessive demands may lead to detachment from one’s work. Excess job demands and insufficient job resources reduce life satisfaction through exhaustion and disengagement/detachment (which together define the experience of burnout) [1]. The JD-R model is commonly used to conceptualize occupational burnout, and its validity has been confirmed in a cohort of nurses [2]. For these reasons, the JD-R model will serve as this study’s conceptual framework for burnout.

Unfortunately, burnout is underrecognized by those who suffer from it, and it typically goes undetected until employees’ performance or health status deteriorates, or other negative consequences of burnout occur. Burnout can be addressed (once it occurs) but can also be prevented from occurring [3, 4]; thus, there is an opportunity for research to predict burnout ahead of its observable, damaging manifestations.

Occupational burnout in healthcare is particularly concerning, given the dependence of the medical system and public health on healthcare workers. According to the World Health Organization, registered nurses (RNs) consist of the majority (59%) of the global healthcare workforce [5]. Despite the essential role of RNs in healthcare delivery, they are especially vulnerable to burnout, given stressful working conditions and occupational challenges that are unique to the nursing field. Indeed, an estimated 35–45% of nurses experience burnout, primarily driven by increased work demands, work inefficiencies, interpersonal conflict, moral distress, and little control over decisions that affect their work [6–13]. Beyond its consequences on the physical and psychological well-being of staff [14], burnout in RNs is associated with an increased risk of hospital-acquired infections, lower quality of care, increased absenteeism, and poor patient satisfaction [6, 15–19]. The importance of reducing the risk of burnout in nurses is further highlighted by the critical shortage of hospital-based RNs in the U.S. and abroad [20]. Burnout-associated turnover in RNs has been associated with high costs to healthcare institutions, estimated at $16,736 per nurse per year employed [21]. While burnout is not a new phenomenon in the nursing field, the additional challenges introduced by the recent COVID-19 pandemic have undoubtedly put additional strain on the healthcare system at large and has primarily affected the frontline nursing population [22–25]. Based on these observations, the early identification of burnout in nurses may lead to more timely intervention on an institutional level before burnout’s harmful and costly effects on nursing professionals, their patients, and healthcare systems surface.

**Fig. 1:**
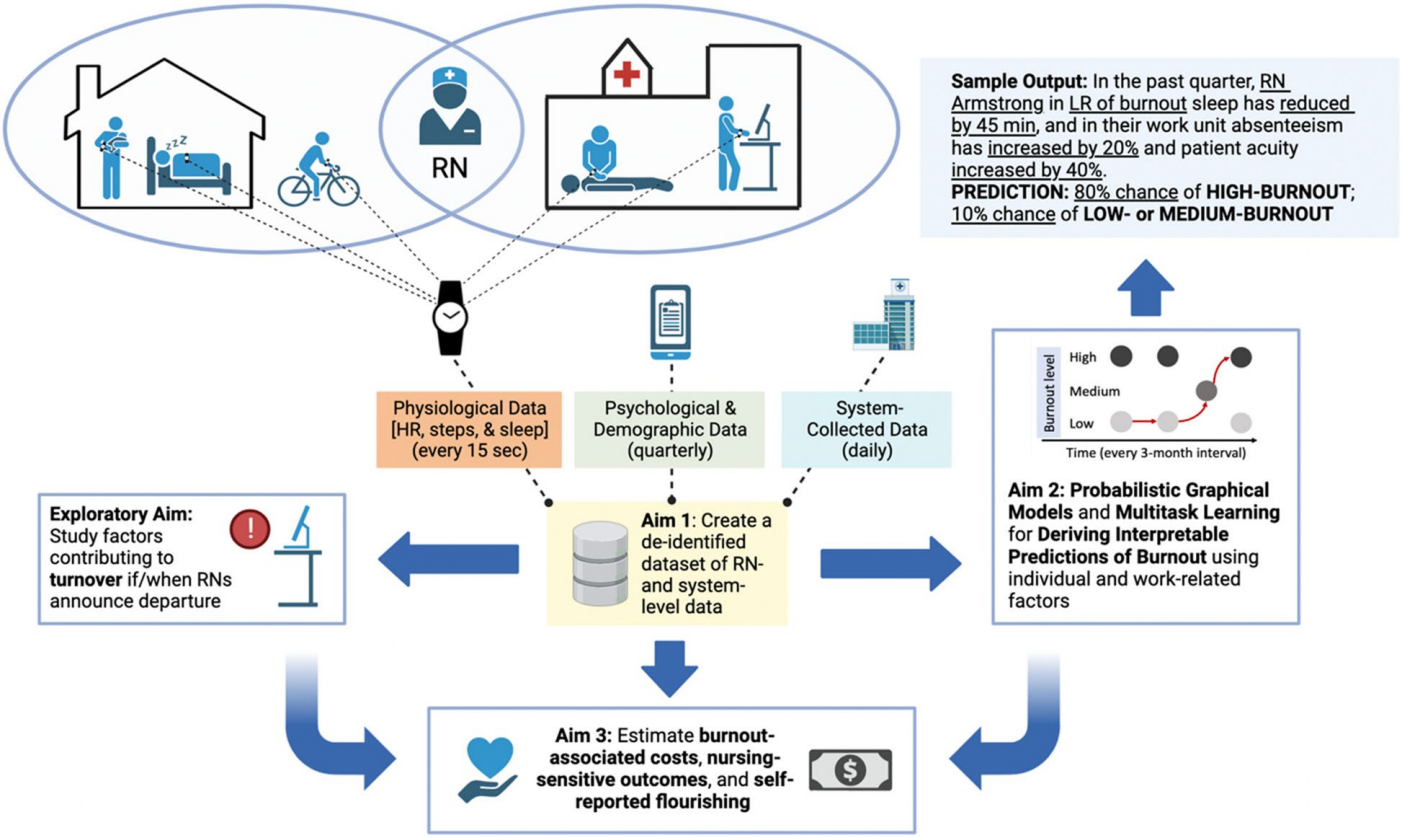
Overview of study aims

Currently, the standard for measuring burnout symptoms is to use a scale, such as the Maslach Burnout Inventory or the Professional Fulfillment Index [6, 14, 26, 27]. These scales capture the severity of burnout symptoms across two or more dimensions (emotional exhaustion, depersonalization, low sense of personal accomplishment), with scores often categorized into low, moderate, or high within each domain or dichotomized into overall burnout versus no burnout. Because most published longitudinal studies of burnout in nursing are primarily survey-driven [28, 29], the possible interaction between psychological and organizational-level factors and physiological correlates of burnout over time has yet to be explored. However, with the capability of real-time digital monitoring of physiology (e.g., heart rate, step count, sleep) using consumer-grade smartwatches, there is an opportunity to create new knowledge on the interaction of physiological, psychological, and workplace factors for the early prediction of impending burnout with the hope of detection before symptoms become noticeable to nursing professionals. The primary outcomes of the BROWNIE study (see Fig. 1) are as follows:

1. A unique, de-identified dataset with 70% completeness will be created that informs the interaction of continuous physiological measures (collected by wearable smartwatches), repeated psychological measures (taken through quarterly surveys), and work-related factors (using administrative databases) for predicting burnout in a diverse sample of hospital-based RNs.
2. An analytical framework combining probabilistic graphical models (PGMs) and multitask learning (MTL) will be developed to derive interpretable predictions of burnout in subsequent cohorts of registered nurses from the dataset generated.

Secondary outcomes of the BROWNIE Study include the estimation of burnout-associated costs, including absenteeism and associated costs, nursing-sensitive outcomes, and self-reported human flourishing across six life domains.

The Burnout PRedictiOn Using Wearable aNd ArtIficial IntelligEnce (BROWNIE) study described in this article hypothesizes that the analytical integration of physiological (from smartwatches), psychological (from rating scales), and workplace factors (from administrative databases) will allow for predicting both individual- and unit-level predictions of burnout prior to its manifestation or worsening using an AI platform.

## Design

The BROWNIE study is a digital health, decentralized, single-arm observational study of registered nurses providing care in hospital-based settings. In allowing for both day- and night-shift RNs, the decentralization of the study allows for broadening study participation to facilitate the study of a broad array of factors potentially associated with burnout. Individual factors will include continuous physiological measures (collected by wearable smartwatches; raw data gathered for every 15-second epoch) and psychological factors (collected quarterly using a soft-touch survey conducted on a web application). Workplace factors (all system-collected) will include patient acuity measures (a proxy for job demand) and nationally reported daily nurse-sensitive patient outcomes. These will include care quality measures (injuries, infections) and patient-perceived satisfaction with care provided. By combining “touch-free” (passively-collected smartwatch data and system-collected work factors) and “soft-touch” (actively-collected online survey) data, this project aligns with the National Academy of Medicine’s recommending regular measurement of burnout without causing additional burden [30].

Three cohorts of RNs will be recruited and observed for 12 months. Baseline information will be collected, and each participant will be provided with a Garmin smartwatch, which will be used to continuously measure physiological parameters relating to stress reactions from job demands/exhaustion (Table 1). Physiological stress-response measurements (collected using smartwatches) will be supplemented with online surveys on psychological symptoms (Table 1), dietary factors (Table 1), workplace exposures (Table 2), and system-collected data (Table 2). Background information will also be collected on sociodemographics, adverse childhood experiences, and basic work exposure information, as shown in Table 3. Specific rating scales and instruments used in this study are summarized in Supplementary Material S1. All surveys will be conducted quarterly using Mayo Clinic’s Survey Research Center via email (which can be accessed remotely beyond the hospital environment) and will take no more than 30 min to complete. System-collected data are automatically collected and will require no effort from study participants.

**Table 1:** Repeated Measures: Rationale and Frequency of Measurements

**Psychological Measures**		
Measurements and Frequency	Data Dimension	Rationale
Maslach Burnout Inventory-Human Services Survey (MBI-HSS), **every quarter**	Burnout risk	• Extensively validated for measuring burnout in nurses • High dimensionality (large number of items useful for predicting burnout)
Center for Epidemiologic Studies Depression Scale (CES-D), **every quarter**	Depressive symptoms	• Validated measure of depressive symptoms
Linear Analogue Self-Assessment (LASA), **every quarter**	Quality of life/well-being	• Validated and multi-dimensional measure of quality of life
Belonging Measure, every quarter	Sense of belonging	• Validated measure of belonging, a concept which is related to burnout
Human Flourishing Index, **final quarter (optional)**	Flourishing	• Validated measure of self-reported flourishing across 6 life domains (happiness/life satisfaction, mental/physical health, meaning/purpose, character/virtue, close social relationships, financial/material stability)
**Physiological Measures**		
Measurements and Frequency	Data Dimension	Rationale
Physiology measures from smart watches, **everyday**	Heart rate variability, sleep duration, sleep quality, activity	• Captures potential physiological effects of stress using variations in activity, sleep, heartrate and/or blood oxygen saturation levels
**Lifestyle Measures**		
Measurements and Frequency	Data Dimension	Rationale
Food intake questionnaire, **every quarter**	Diet	• Captures potential dietary associations with stress, as some elements of diet have been previously associated with burnout symptoms^51^
**Work-related Measures**		
Measurements and Frequency	Data Dimension	Rationale
Catheter-Associated Urinary Tract Infections [CAUTI], Central Line Associated Blood Stream Infections [CLABSI] and Patient falls, **system-collected and nationally-reported everyday **	Infection prevention and control	• Most-common nurse-sensitive adverse patient outcomes, which institutions strive to reduce to achieve nursing quality accolades • Burnout in RNs associated with low-quality care
4 Nurse-sensitive items in the Hospital Consumer Assessment of Healthcare Providers and Systems (HCAPHS) survey, **system-collected and nationally-reported everyday**	Patient satisfaction of care provided by nursing staff	• Nurse burnout is associated with lower quality of care provided • Patient satisfaction levels are lower in hospitals where burnout rates are high
Nurse-sensitive workplace exposures (see Table 1b), **system-collected every quarter**	Workplace exposures	• Burnout associated with workplace related exposures

**Table 2:** Additional workplace exposures (Yes: y; No: n)

**System-Collected**		**Participant Reported**	
Number hours worked (from timesheets)	Absent Days (y/n) (not vacation or holiday)	Conflicts with patients (y/n)	Involvement in decisions about withdrawing treatment (y/n)
Number overtime hours worked	Vacations or holidays (y/n)	Conflicts with patient family members (y/n)	Discussing bad news about diagnosis/prognosis with family members (y/n)
Night shifts (y/n)	Exposure to patient death (y/n)	Conflicts with colleagues (y/n)	Discussing bad news about diagnosis/prognosis with patients (y/n)
Extra shifts (y/n)	Number of deceased patients	Involvement in decisions about withholding treatment (y/n)	Involvement in situation(s) that caused moral distress (y/n)
Patient Acuity	Full-Time Effort (FTE; 0 for 0 hours, 1 for 40 hours/week)	Conflicts with superiors (y/n)	Conflicts with other professionals (y/n)

**Table 3:** Demographic, clinical and work exposure information

**Baseline demographic and clinical information**		**Basic work exposure information**
Age at enrollment	Highest education level	Primary work unit (medical/surgical unit, ICU, ER, obstetrics, OR/recovery room, pediatrics, neonatal ICU)
Sex, Race, Ethnicity (Hispanic, Non-Hispanic)	Number of children	Secondary work unit (if applicable)
Marital status	Subjective pain rating	Primary patient population
Diagnosed chronic medical conditions and substance exposures	Diagnoses of mental health conditions	Employment type (permanent, temporary)
Treatment status of mental health conditions	Adverse childhood experiences (ACE)^52^	Years of RN experience at institution
		Years of RN experience (total)

## Methods

### Recruitment

**Fig. 2:**
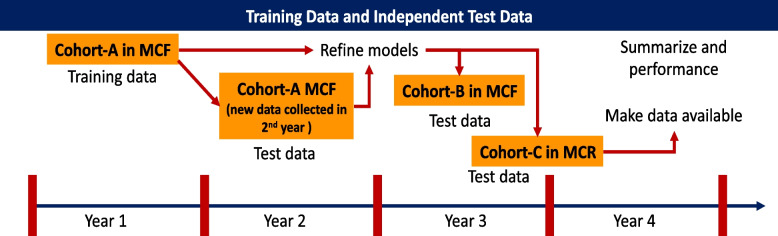
Training and Testing Cohorts. Cohort-A (*n*=120) will be recruited in Year 1. Data from Cohort-A will be used to train a machine learning model for predicting burnout. Cohort-A will continue to provide data for first 6 months of 2nd year) in 2^nd^ and 3rd quarter of Year 2 for testing prediction models. Cohort-B (*n*=120) will be recruited in Year 3 to validate and refine the probabilistic graphs developed using Cohort A. Cohort-C (*n*=120) will be recruited in mid-Year 3 as an external validation cohort

**Fig. 3:**
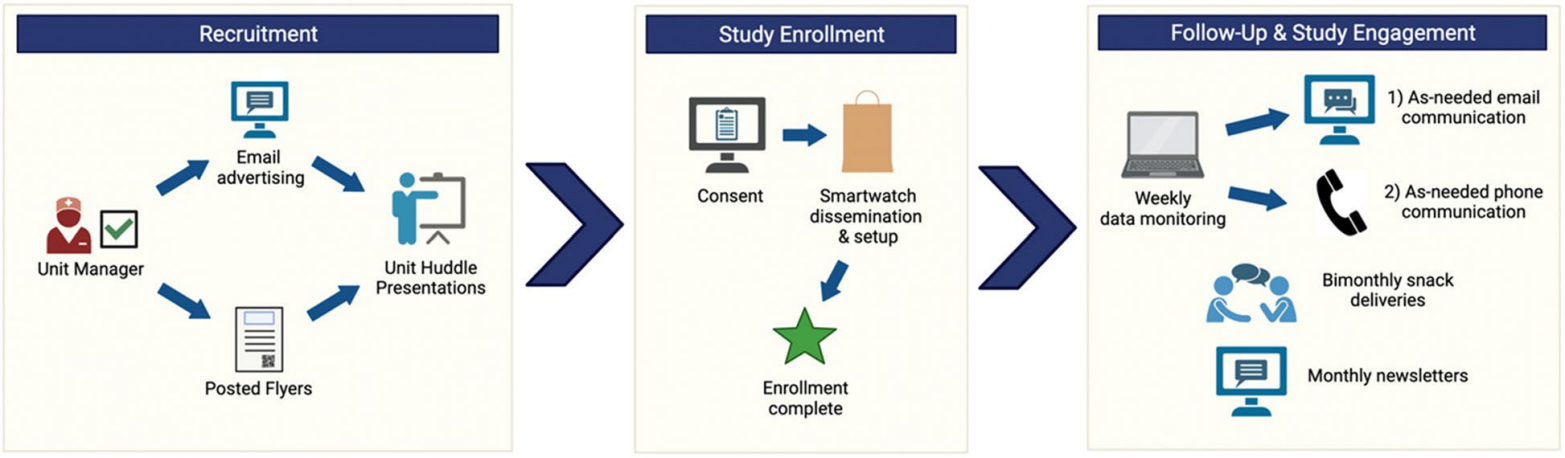
Study communication and engagement strategy

 Three cohorts of 120 RNs will be recruited from Mayo Clinic (total of *N* = 360). Each cohort will comprise RNs from the Emergency Department, Intensive Care Units (including neonatal), selected Medical/Surgical units, and Inpatient Psychiatry. As summarized in Fig. 2, Cohort-A (*n* = 120) will be recruited in Year 1. Data from Cohort-A will be used to train a machine-learning model for predicting burnout. Participants in Cohort-A will be reconsented to contribute data during Year 2 to refine the prediction models developed using the year-long data from Cohort-A. Cohort-B (*n* = 120) will be recruited in Year 3 to test the models. Finally, Cohort-C (*n* = 120) will be recruited in mid-Year 3 as an external validation cohort to evaluate the accuracy of the burnout models in a separate population. In partnership with nursing leadership, trained research staff will recruit potential study participants by email using administrative rosters, which contain information on primary work units assigned and email addresses. Research staff will also liaise with nursing management teams for more informal recruitment efforts. These efforts will include advertising by internet (Mayo Clinic intranet) and posting study advertising flyers. Potential study participants will attend an oral presentation at work unit meetings where research staff will explain the study’s aims and eligibility criteria, summarize the research procedures, and address questions or concerns about study participation. Individuals who express interest in and are deemed eligible for study participation will provide digitally based informed consent via email. The outline of the recruitment and consent process is illustrated in Fig. 3.

### Inclusion/Exclusion criteria

Eligible subjects at baseline will be RNs employed at Mayo Clinic in the Rochester, MN or Jacksonville, FL locations, male or female, aged 20–70 years on the date of screening, who are employed full-time or part-time (≥ 60% of full-time effort). Eligible RNs will be hospital-based, meaning that their primary work assignment will be medical-surgical units, operating rooms, emergency room, psychiatric, intensive/cardiac care units, or other special care units (including neonatal intensive care units and newborn nurseries, or advanced care at home) and have an Android or iOS smartphone. Proficiency in written and spoken English, and the ability to provide valid, informed electronic consent (e-consent), will be required for study participation. If an existing participant terminates their employment at Mayo Clinic but continues employment as a hospital-based RN at another institution, they can complete their full term in the study. However, system-collected data will not be obtained from their new institution.

### Smartwatches, dissemination, and decentralized setup

After obtaining e-consent, study subjects will be issued a Garmin vivoactive4 smartwatch (or its updated model at the time of issue) and a study-developed enrollment manual; the items will be delivered to their work unit to minimize oversight and contact with research personnel during patient care activities (see Fig. 3). The manual will contain instructions on the use and basic maintenance of the smartwatches alongside research staff contact information. Subjects will independently follow the study-provided setup manual to enroll in Garmin Connect (a mobile application designed by Garmin that collects physiological data from the smartwatches) and Fitabase (an online data management platform that pulls data from the subject’s Garmin Connect online account); trained research staff will de-identify subjects on the Fitabase platform (subject ID and anonymized number ID maintained by PIs). Should questions arise about the basic operation and maintenance of the smartwatches, subjects will be informed that research staff can be contacted anytime.

### Data collection

#### Data collection from smartwatches

Garmin vivoactive4 (or an updated model) smartwatches will provide continuous health monitoring features to keep track of energy levels, pulse oximetry, respiratory rate, stress levels, sleep patterns, and heart rate while operating with an 18-hour battery life per charge cycle. A parser has been designed to query Fitabase every 14 days to download the raw Garmin Connect data and process it for analyses by summarizing the sensor features by minute, hour, day, week, or month. All data will be downloaded and structured within Mayo Clinic research storage systems managed by institutional Information Technology teams.

#### Data from psychological, dietary, and workplace exposure surveys

Electronic surveys (see Tables 1, 2, and 3) will be provided quarterly to all cohort participants. The Mayo Clinic Survey Research Center will send quarterly assessments to subjects through their institutional email addresses. Survey data will be stored in a REDCap database (managed by the Survey Research Center), password-protected, and accessible only by authorized research staff. Following weekly reminders sent by the Mayo Clinic Survey Research Center, trained research staff will follow up with participants via telephone if surveys are not completed after 4 weeks.

#### System-collected data

System-collected data are regularly reported by the Mayo Clinic institution daily. Several of these measures are considered nursing quality indicators that are reported to the Centers for Medicare and Medicaid Services (CMS) as part of their Hospital Consumer Assessment of Healthcare Providers and Systems (HCAPHS) survey. Since these data are collected and stored electronically by the institution (administrative databases and electronic timesheets), these data elements do not need to be collected from participants. These data are aggregated at the unit level. Specific system-collected data elements are listed in Table 1 and Table 2. This information will be manually abstracted from the Mayo Clinic Quality Management Portal and stored in a password-protected study database.

#### Collection of patient acuity measures

Daily patient acuity measures will be collected from the participating nursing units for the study period. This data is collected via the Harris Healthcare Acuity Plus system. Finally, the number of patient deaths on the units during the study timeframe will be identified through nursing administration.

#### Collection of productivity measures

Administrative records will be used to define two organizational productivity outcomes: absenteeism and reduced Full Time Effort (FTE). FTE at Mayo Clinic is calculated biweekly based on full-time requirements and is standardized across units. This data will be manually abstracted from Kronos Workforce Timekeeper, the timekeeping software used by the institution. This software stores data from nurses’ timecards that are tabulated each pay period. Data collected will include the number of hours participants worked, unplanned absent days (excluding vacations or holidays), overtime hours, hours over their FTE, and night shifts worked.

### Plan for establishing predictability of burnout

**Fig. 4:**
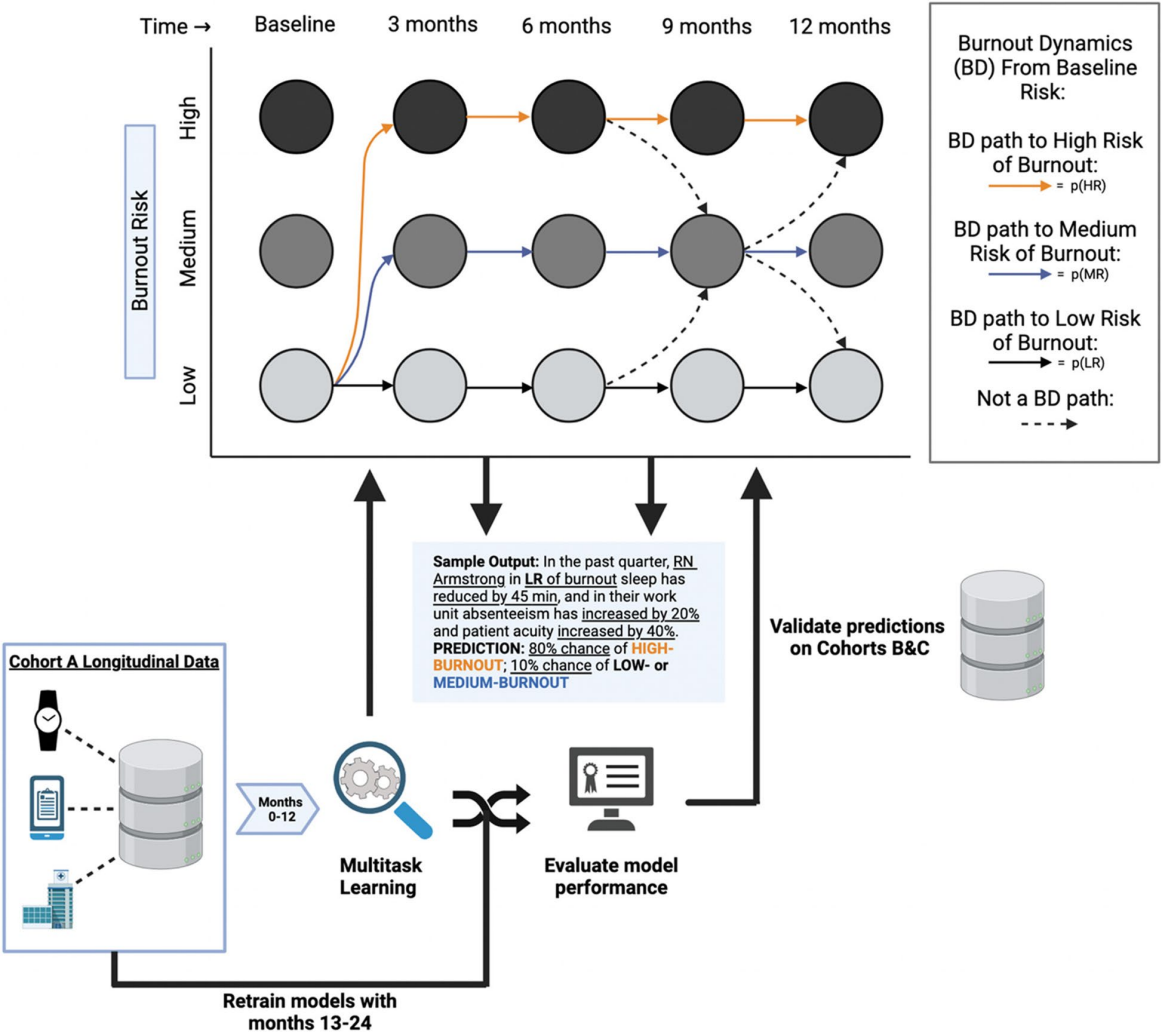
Analytical approach overview

Developing a prediction model for burnout using Aim 1 data will proceed in two phases. For Phase 1, probabilistic graphical modeling will be used to establish Burnout Dynamics (BD). Burnout dynamics (the most likely variations in burnout over time) will be derived using dynamic Bayesian networks (DBN) – which subsume hidden Markov models [31, 32]. A DBN comprises states (nodes) and probabilistic transitions between states (see Fig. 4) [33]. There are three hidden (latent) states represented by the risk of high burnout defined by the MBI (defined by Martins Pereira et al. (2016)), namely high risk (HR), medium risk (MR), and low risk (LR) at each time point of the study (each time point being one of the 4 quarters of the 12-month study). Transition probabilities between two latent states from three consecutive time points will be the fraction of subjects from the origin state transitioning to a given latent state at the next timepoint. Burnout Dynamics are those trajectories with the highest likelihood starting from a burnout risk (e.g., low) at baseline (start of study) and to any of the three burnout risk levels (e.g., high) having sustained that burnout severity for two consecutive quarters (see Fig. 4).

For Phase 2, data from year 1 of Cohort-A will be used to train MTL models to estimate the risk of burnout (multiple tasks referring to high, medium, and low) in the next quarter (see Fig. 2). Models will then be tested and retrained using data from 13 to 24 months of Cohort-A. Predictions will then be evaluated in Cohorts B and C. Standard model performance metrics (e.g., AUC, sensitivity, specificity) will be reported. Predictor variables will be ranked by the relative importance in the prediction models, and the top 10 predictors (a heuristic for simplicity in deriving easily interpretable predictions in English sentences) will comprise Core Burnout Factors. As there are no established benchmarks for predicting burnout, predictive accuracies > 70% will be aimed for, which is clinically acceptable for psychiatric disorders [34–36]. The input features for training classifiers will start at a given burnout risk level (e.g., High Risk) and BD paths.

If subjects are not equally distributed across 3 risk levels, burnout risk will be dichotomized into “high” and “low.” There will likely be sufficient distribution given that an estimated 35–45% of nurses of RNs report burnout [6–13]. In that case, PGMs will be developed with two burnout states for each time point.

### Estimate burnout-associated cost in nurses due to lost productivity

A conservative cost-consequence analysis will be conducted using data from 3 nursing cohorts across 4 nursing units (Emergency Department, Intensive Care Units [including neonatal], selected Medical/Surgical, and Inpatient Psychiatry). The analysis will focus on two organizational outcomes: absenteeism and reduced FTE. These outcomes directly reflect on institutional nursing capacity, which is a vital consideration nationally (from a public health policy perspective) and institutionally (from a managerial perspective). The study is deemed conservative because other burnout-related costs (e.g., reputational impact from poor patient outcomes, hiring/training RNs) will be omitted.

Input parameters for this analysis will be: (i) burnout risk status among study RNs based on quarterly MBI scores (high-risk), (ii) actual absent days attributable to burnout per quarter, (iii) actual reduction in FTE attributable to burnout per quarter (including turnover where FTE becomes 0), and (iv) median compensation for 1.0 FTE in RN units (to derive costs from absent days). The primary output will be the cost attributable to burnout per nurse per quarter, defined as the difference in costs attributable to absent days and reduced FTE in those with high- or medium-risk vs. low-risk of burnout (risk levels defined by the MBI). Sensitivity analyses will be conducted to assess the effect of (i) associating demographic factors and odds-ratio of burnout (within the nursing unit) with cost estimates, (ii) nursing units on cost estimates of burnout (i.e., cost estimates in ICU vs. inpatient psychiatry), and (iii) location on cost estimates of burnout at unit-level between geographical sites (i.e., ICU in MCF vs. MCR).

### Power calculations

For achieving statistical power of 0.7 and a 2-sided alpha level of 0.05, accommodating 15% drop-out (including smartwatch loss, job turnover, or damage to smartwatch) and potential class imbalance of 1:3 (~ 30% burnout in RN staff), the required sample size is 118 participants per cohort of the study. We anticipate meeting this sample size requirement in this project based on eligible RN staff at both sites. As of 2022, the Mayo Clinic Rochester and Florida campuses employed 1916 and 449 RNs, respectively, that met the study’s eligibility criteria.

## Limitations, risks, and alternative strategies

### Study drop-out mitigation strategies

To satisfy the proposed study aims, participating RNs will be encouraged to wear the smartwatch; nevertheless, there is still a risk of nurses not continuously wearing the smartwatch. Thus, remuneration will be offered for both surveys and wear-time as a drop-out mitigation strategy. In compliance with institutional policies, subjects will be remunerated up to $225 for the study (pro-rated and payable after completion of each survey: $25–1st quarter, $50–2nd and 3rd quarter, and up to $100–4th quarter, accommodating for the 12 optional additional survey items associated with the Human Flourishing Index) and they will get to keep the study-issued smartwatch. After completing the 4th quarter assessment measures, participants will be invited to take the optional Human Flourishing Index for an additional remuneration of $25 on top of the $75 remuneration for the 4th quarter assessment. The investigator team will also communicate a wear-time goal of 70% to participants and offer an additional $20 for maintaining a 70% or greater wear-time.

### Decentralized follow-up and engagement strategy

Follow-up will begin on the date of the baseline study visit and will end at 12 months following the beginning of follow-up. Wear-time statistics will be collected via Fitabase and will be closely monitored. If a subject has not synced their watch data onto the Garmin Application in over 7 days or their wear time is below 70% over a 7-day average, the subject will receive an email from study personnel reminding them to resume wear and sync their watch data on the Garmin Application. If the investigator team sees no improvement over the following 7-day period, the participant in question will receive a phone call from study personnel to their work unit when they are on shift. If technical issues or discomfort are communicated, the study team will work to resolve the issues. If they do not comply for an additional 14 days, the participant will receive a call from the study PI, and a decision will be made as to whether the participant will be disengaged from the study.

Snacks will be delivered to participating units every two weeks to promote engagement. An investigator team member will be present during that delivery to engage with participants, encourage regular synchronization of watch data, remind participants to complete quarterly surveys, and answer any questions that participants may have to establish trust and rapport. Watch charging stations will also be provided in the breakrooms of participating units so participants on shift can charge the watches.

As an additional engagement strategy, monthly newsletters will be dispersed to study participants wherein study progress, aggregate data collected (e.g., cumulative statistics on sleep, hours of data, and wear-time), and sample statistics (e.g., unit-level total numbers) are reported. Participants’ experiences may also be featured (e.g., a positive experience with the watch or an anecdote relating to the study) only if they consent to sharing. Additionally, smartwatch features of interest will be included in the newsletter to encourage participants to use their devices. The newsletters will be approved by Mayo Clinic IRB prior to dissemination. The overview of the study’s engagement strategy is illustrated in Fig. 3.

### Anticipation and plan for turnover

Turnover is defined as the entry and exit of employees from an organization or unit. Participating RNs may transfer to a different unit, move to a different organization, or quit/change their profession throughout the study. According to a recent survey, 23% of RN respondents indicated intent to leave their current position within 6 months [22]. Various professional factors contribute to nurse turnover, including burnout, role perceptions, management style, career advancement, and employment benefits. Personal factors such as health problems, finances, and familial responsibilities also contribute to nurse turnover. Nurse turnover negatively impacts patient care outcomes, patient satisfaction, morale of the unit, and is costly [37]. Because of the risk of RN turnover during the study, there is a plan to incorporate the RNs that quit working at the recruiting institution but still maintain nursing-based jobs outside of the institution and a plan to perform secondary analysis on that data to study factors contributing to turnover.

When the investigator team is informed about a study RN’s turnover prior to completion of the study, provided that the RN will be continuing in the nursing profession and the study RN is willing to continue to share smartwatch and survey data for the remainder of the study duration, the investigator team will seek additional consent from the participant. Upon consent, a brief survey will be administered to document the factors associated with turnover (see Supplementary Material S2 for turnover assessments). Should the participant consent to the turnover study, they will participate with the existing smartwatch.

### Potential factors that might limit MTL success

Given that this is the first exposition of MTL for predicting burnout, common burnout factors may not be identified that simultaneously improve the predictability of burnout. ***Alternative strategy:*** If tasks cannot be learned simultaneously using one model, multiple single-task predictive models will be trained (for each burnout level), and then a consensus of how different tasks are biased by the ranks of predictor variables will be constructed. Confidence that this limitation can be overcome with the proposed alternative strategies has been reached by the investigator team with the current estimate of sample availability.

### Study limitations

This proposed study has several limitations. There is a chance that bias could be introduced in future prediction modeling due to sampling limitations. The eligibility criteria for the study do not include any screening for pre-existing burnout. Because eligible participants volunteer to join the study, there is a chance that our recruited sample could consist of predominantly less burned-out individuals than the general population of RNs. Additionally, because system-level data is taken at a unit level at our institution and does not include performance metrics of individual RNs, sampling was logistically limited to entire units. Due to this limitation, the sample is inherently not independent. Participants must work with one another, which may influence burnout in one another. To address this limitation in our analyses, the study team asks quarterly questions to address participants’ perceptions of unit dynamics, including a belongingness questionnaire and questions about workplace conflicts.

### Participant confidentiality

All participants will be assigned a unique study subject ID number, and all study data will be maintained on a password protected database accessible only by IRB-approved research staff. Data collected from smartwatches and psychological measures will be linked to each subject ID number. To achieve the analytic aims described above, inputs for data analysis will include subject ID number, subject sex, data collected from smartwatches, and scores on psychological rating instruments. No protected health information will be entered into the study databases used for data analysis, such as subject names, addresses, telephone numbers, social security numbers, Mayo Clinic patient numbers, or any other data that could serve as identifiers. A separate file containing protected health information will be stored in a password-protected electronic file to which only the PIs and select study personnel have access.

### Premature discontinuation from the study

Study participants may discontinue participation at any time. An administrative entry indicating discontinuation of participation will be made into the study database in these cases.

A subject may be withdrawn by the study investigator(s) for any of the following reasons:

- Loss to follow-up.
- Withdrawal of consent.
- Death.
- Violation of protocol procedures, as per investigators’ judgment.
- The investigator believes it is in the best interest of the subject to discontinue the study (e.g., for safety or tolerability reasons).

If a subject withdraws from the study, s/he can request a visit with a study investigator to discuss their reason(s) for withdrawal. Study data that have already been collected will be retained and used in accordance with the subjects’ original informed consent. The subject may withdraw consent to use this data, in which case the subjects’ data will not be used for analysis.

### Behavioral health risk management plan

The enrollment for this study is not targeting persons with mental health conditions, and this study is not a clinical intervention study. Nevertheless, behavioral health issues and even emergencies may arise during the study.

Potential participants will be notified in the consent form that: “This study includes the assessment of depression symptoms; however, the study staff will not be actively monitoring for depression, treating depression, or referring you for treatment. If you experience depression, thoughts of suicide, or any other mental health concern during the study, you will be responsible for arranging for medical care.”

If a study participant notifies any research staff of having suicidal ideation or thoughts of wanting to end their life, the participant will be evaluated the same day by a research study clinician for further consideration of study participation and management of suicide risk according to standard of care practices.

### Common adverse effects

The study procedures are not anticipated to result in the frequent (or common) occurrence of adverse effects. There may be some mild discomfort from wearing smartwatches that will be discussed with participants, although continuous use with breaks (e.g., during showering) will be encouraged. Some participants may also experience mild anxiety, fatigue, or emotional discomfort when answering behavioral questions on the brief questionnaires. To reduce these concerns, the frequency of completing these questionnaires has been minimized.

## Discussion

### Future implications

The use of digital methods to study burnout in healthcare employees has previously surfaced in the context of deploying wearable technology to investigate correlates linking to burnout in nurses and other healthcare professionals [38, 39]. However, due to being short in length, prior studies have yet to account for seasonal changes in work conditions and the long-term manifestation of burnout. Most preexisting studies also have only collected data during work shifts, which could omit personal and lifestyle factors that could influence burnout syndrome.

In addition to addressing these limitations, this protocol introduces a novel method of prospectively predicting burnout phenomena using the continuous physiological measures taken from the wearable devices over a year-long period and combining the psychological and workplace-related factors that may forecast burnout in real time. Predicting a burnout syndrome prospectively – instead of retrospectively analyzing data to look for specific correlates of burnout syndrome - could enable a future institutional response within a more realistic dynamic environment to intervene early and prevent burnout before it occurs. Moreover, this methodology renders an augmented, comprehensive approach to burnout prediction where workplace-wide and institutional-level factors are also considered in addition to individual physiological and psychological measures. The consideration of a variety of external factors in our future analysis helps avoid viewing the individual as solely responsible for their burnout.

While burnout syndrome remains a consistent issue in the nursing population, burnout is prevalent in every workplace [14, 40]. Thus, these methods could be potentially adjusted and applied to study other healthcare workers (e.g., clinicians) and may translate to non-healthcare workforces despite being designed to predict burnout in RNs. If successful, this project will be an important first step toward the use of smart devices as an aid in forecasting burnout risk in real time.

### Decentralized procedures

The nursing population is commonly met with obligations, including existing patient care tasks, administrative assignments, and other duties. In addition to these obligations, the exigencies and risks stemming from the COVID-19 pandemic could cause subjects to perceive day-to-day study processes as an additional burden [23]. Furthermore, the variability of nursing schedules further complicates in-person study operations. For example, participants working night-shift schedules will have increased difficulty completing in-person study activities that will typically occur during the day. Considering the study’s primary outcome to create a dataset that accounts for the variability in the hospital-based RN population, the study protocol has been designed to decentralize study operations. The protocol was designed to limit face-to-face interaction with research personnel as much as possible. To this end, most recruitment presentations will be held online (via video conference system) at routine nursing huddle meetings. These meetings will be recorded for eligible nurses who may have been absent. Communications with participants will be held by institutional email and phone unless a face-to-face visit is requested.

The consent and enrollment process has also been designed to be remote and asynchronous, as observing this approach enables the study team to deliver their study device and materials to each subject discreetly. Subjects can consent, set up their devices on their own schedules, and have easy access to research team contact information should they need assistance. All electronic participant-reported outcome (ePRO) surveys will be administered online and set to automatically send to participants through their institutional email, with responses stored in an institutional survey research database. Aside from any in-person visits to help with technical support aspects of the watch (e.g., change in settings), monitoring of study participation and follow-up will also be remote. The development of a remote, decentralized process for the study will both minimize time away from patient care and allow for inclusivity of several nursing populations who may work irregular schedules and in varying settings in accordance with the study’s primary outcomes.

### Addressing compliance

As completeness of the dataset is integral to the validity of future prediction models that will be generated as the study’s primary outcomes, the integrity of the models generated from this study relies on participants wearing their devices. For this reason, along with the study’s decentralized design, length, and population, ample consideration was given to how the investigator team would engage with participants to minimize attrition and increase compliance. A 2022 systematic review of human studies involving a wearable device found that the most common study length for participation involving wearing the sensor is one week [41]. Conversely, a year was chosen as the length of participation in this study because burnout is poorly understood in its etiology over time, and seasonal changes may influence the working conditions of hospital-based RNs.

Wear-time compliance is a general challenge for studies involving fitness trackers or similar devices [42–44]. Notably, wearables studies that are longer in duration can have compliance rates as low as 16% [45]. Factors contributing to reduced compliance in wearables studies include technical issues, personal reasons, employment reasons, survey burden, and setup difficulties [45, 46], with technical issues being a primary concern. In an effort to circumvent technical challenges, the study team will monitor smartwatch data weekly, and as needed, email reminders will be sent to participants systematically if data is not being received. Participants can expect a courtesy call a week after they receive a reminder email. Therefore, should technical problems arise, they can be addressed promptly to minimize data loss and participant frustration. Martinez et al. also identified survey burden as a factor that could inhibit wear-time compliance, so to reduce this risk, ePRO surveys have been arranged to be completed at quarterly intervals such that study participation is primarily passive monitoring. Additionally, the twice-monthly snack deliveries serve as both a retention strategy and a means for participants to voice concerns about any challenges and receive assistance from the investigator team.

An added consideration for increasing compliance was the choice of the smartwatch device. The smartwatch in the study was chosen partly because of access to physiological data from the device, 5-day battery life, and compatibility with both iOS and Android operating systems (to reduce sampling bias based on phone vendors). However, in addition to these logistical considerations, the investigator team purposefully picked a consumer-grade smartwatch with features and aesthetics comparable with devices RNs, as general consumers, would more likely purchase for personal use and, therefore, would be less stigmatizing in the workplace. This choice of device is also believed to influence compliance as it is reasonable that the RNs will consistently wear the devices over a year-long period if they enjoy using and wearing them.

### Supplementary Information

**Additional file 1.**

## Data Availability

Not applicable.

## Abbreviations

RN: Registered Nurse
BROWNIE: Burnout PRedictiOn Using Wearable aNd ArtIficial IntelligEnce
FTE: Full-Time Effort
MTL: Multi-Task Learning
MBI: Maslach Burnout Inventory
DBN: Dynamic Bayesian Network
BD: Burnout Dynamics
ICD-11: The 11th Revision of the International Classification of Diseases
AI: Artificial Intelligence
CMS: Centers for Medicare and Medicaid Services
HCAPHS: Hospital Consumer Assessment of Healthcare Providers and Systems
LR: Low Risk
MR: Medium Risk
HR: High Risk
PGM: Probabilistic Graphical Model
ICU: Intensive Care Unit
HIPAA: Health Insurance Portability and Accountability Act
ePRO: Electronic Participant-Reported Outcome
MCR: Mayo Clinic Rochester
MCF: Mayo Clinic Florida

## References

[1] Demerouti E, Bakker AB, Nachreiner F, Schaufeli WB (2001). The job demands-resources model of burnout. J Appl Psychol.

[2] Demerouti E, Bakker AB, Nachreiner F, Schaufeli WB (2000). A model of burnout and life satisfaction amongst nurses. J Adv Nurs.

[3] West CP, Dyrbye LN, Shanafelt TD (2018). Physician burnout: contributors, consequences and solutions. J Intern Med.

[4] Zhang XJ, Song Y, Jiang T, Ding N, Shi TY (2020). Interventions to reduce burnout of physicians and nurses: an overview of systematic reviews and meta-analyses. Med (Baltim).

[5] Organization WH. State of the world’s nursing 2020: investing in education, jobs and leadership. In.; 2020. https://www.who.int/publications/i/item/9789240003279.

[6] Aiken LH, Clarke SP, Sloane DM, Sochalski J, Silber JH (2002). Hospital nurse staffing and patient mortality, nurse burnout, and job dissatisfaction. JAMA.

[7] Cimiotti JP, Aiken LH, Sloane DM, Wu ES (2012). Nurse staffing, burnout, and health care-associated infection. Am J Infect Control.

[8] Laschinger HK, Wong CA, Greco P (2006). The impact of staff nurse empowerment on person-job fit and work engagement/burnout. Nurs Adm Q.

[9] Leiter MP, Maslach C (2009). Nurse turnover: the mediating role of burnout. J Nurs Manag.

[10] McHugh MD, Kutney-Lee A, Cimiotti JP, Sloane DM, Aiken LH (2011). Nurses’ widespread job dissatisfaction, burnout, and frustration with health benefits signal problems for patient care. Health Aff (Millwood).

[11] Stone PW, Du Y, Gershon RR (2007). Organizational climate and occupational health outcomes in hospital nurses. J Occup Environ Med.

[12] Vahey DC, Aiken LH, Sloane DM, Clarke SP, Vargas D (2004). Nurse burnout and patient satisfaction. Med Care.

[13] Woodhead EL, Northrop L, Edelstein B (2016). Stress, Social Support, and Burnout among long-term care nursing staff. J Appl Gerontol.

[14] Salvagioni DAJ, Melanda FN, Mesas AE, González AD, Gabani FL, Andrade SM (2017). Physical, psychological and occupational consequences of job burnout: a systematic review of prospective studies. PLoS ONE.

[15] Fida R, Laschinger HKS, Leiter MP (2018). The protective role of self-efficacy against workplace incivility and burnout in nursing: a time-lagged study. Health Care Manage Rev.

[16] Holden RJ, Scanlon MC, Patel NR, Kaushal R, Escoto KH, Brown RL, Alper SJ, Arnold JM, Shalaby TM, Murkowski K (2011). A human factors framework and study of the effect of nursing workload on patient safety and employee quality of working life. BMJ Qual Saf.

[17] Meeusen VC, Van Dam K, Brown-Mahoney C, Van Zundert AA, Knape HT (2011). Understanding nurse anesthetists’ intention to leave their job: how burnout and job satisfaction mediate the impact of personality and workplace characteristics. Health Care Manage Rev.

[18] Parker PA, Kulik JA (1995). Burnout, self- and supervisor-rated job performance, and absenteeism among nurses. J Behav Med.

[19] Spence Laschinger HK, Leiter MP (2006). The impact of nursing work environments on patient safety outcomes: the mediating role of burnout/engagement. J Nurs Adm.

[20] Drennan VM, Ross F (2019). Global nurse shortages-the facts, the impact and action for change. Br Med Bull.

[21] Muir KJ, Wanchek TN, Lobo JM, Keim-Malpass J (2022). Evaluating the costs of nurse burnout-attributed turnover: a Markov modeling approach. J Patient Saf.

[22] Association AN. COVID-19 Impact Assessment Survey—The Second Year. In.; 2022.

[23] Clari M, Luciani M, Conti A, Sciannameo V, Berchialla P, Di Giulio P, Campagna S, Dimonte V. The impact of the COVID-19 pandemic on nursing care: a cross-sectional survey-based study. J Pers Med. 2021;11(10):945. 10.3390/jpm11100945 PMC8538569 34683086

[24] Galanis P, Vraka I, Fragkou D, Bilali A, Kaitelidou D (2021). Nurses’ burnout and associated risk factors during the COVID-19 pandemic: a systematic review and meta-analysis. J Adv Nurs.

[25] Sriharan A, West KJ, Almost J, Hamza A (2021). COVID-19-Related Occupational Burnout and Moral Distress among nurses: a Rapid Scoping Review. Nurs Leadersh (Tor Ont).

[26] Schonfeld IS, Bianchi R, Palazzi S (2018). What is the difference between depression and burnout? An ongoing debate. Riv Psichiatr.

[27] Wirtz PH, von Känel R (2017). Psychological stress, inflammation, and Coronary Heart Disease. Curr Cardiol Rep.

[28] Fornes-Vives J, Garcia-Banda G, Frias-Navarro D, Pascual-Soler M (2019). Longitudinal study predicting burnout in Spanish nurses: the role of neuroticism and emotional coping. Pers Indiv Differ.

[29] Guastello AD, Brunson JC, Sambuco N, Dale LP, Tracy NA, Allen BR, Mathews CA. Predictors of professional burnout and fulfilment in a longitudinal analysis on nurses and healthcare workers in the COVID-19 pandemic. J Clin Nurs. 2022. 10.1111/jocn.16463. Advance online publication. 10.1111/jocn.16463 PMC9538120 35949164

[30] National Academies of Sciences E, Medicine. National Academy of M, Committee on Systems Approaches to Improve Patient Care by Supporting Clinician W-B. In: *Taking Action Against Clinician Burnout: A Systems Approach to Professional Well-Being* edn. Washington (DC): National Academies Press (US) Copyright 2019 by the National Academy of Sciences. All rights reserved.; 2019.

[31] Ghahramani Z. Learning dynamic Bayesian networks. *Adaptive Processing of Sequences and Data Structures: International Summer School on Neural Networks ER Caianiello Vietri sul Mare, Salerno, Italy September 6–13, 1997 Tutorial Lectures* 2006:168–197.

[32] Oliver N, Horvitz E. A comparison of hmms and dynamic bayesian networks for recognizing office activities. In: *User Modeling* 2005: 10th International Conference, UM 2005, Edinburgh, Scotland, UK, July 24–29, 2005 Proceedings 10: 2005: Springer; 2005: 199–209.

[33] Koller D, Friedman N (2009). Probabilistic graphical models: principles and techniques.

[34] Iniesta R, Hodgson K, Stahl D, Malki K, Maier W, Rietschel M, Mors O, Hauser J, Henigsberg N, Dernovsek MZ (2018). Antidepressant drug-specific prediction of depression treatment outcomes from genetic and clinical variables. Sci Rep.

[35] Iniesta R, Malki K, Maier W, Rietschel M, Mors O, Hauser J, Henigsberg N, Dernovsek MZ, Souery D, Stahl D (2016). Combining clinical variables to optimize prediction of antidepressant treatment outcomes. J Psychiatr Res.

[36] Iniesta R, Stahl D, McGuffin P (2016). Machine learning, statistical learning and the future of biological research in psychiatry. Psychol Med.

[37] Bae SH (2022). Noneconomic and economic impacts of nurse turnover in hospitals: a systematic review. Int Nurs Rev.

[38] Feng T, Booth BM, Baldwin-Rodríguez B, Osorno F, Narayanan S (2021). A multimodal analysis of physical activity, sleep, and work shift in nurses with wearable sensor data. Sci Rep.

[39] Kaczor EE, Carreiro S, Stapp J, Chapman B, Indic P (2020). Objective measurement of physician stress in the Emergency Department using a Wearable Sensor. Proc Annu Hawaii Int Conf Syst Sci.

[40] Duke NN, Gross A, Moran A, Hodsdon J, Demirel N, Osterholm E, Sunni M, Pitt MB (2020). Institutional factors Associated with Burnout among Assistant professors. Teach Learn Med.

[41] Chan A, Chan D, Lee H, Ng CC, Yeo AHL (2022). Reporting adherence, validity and physical activity measures of wearable activity trackers in medical research: a systematic review. Int J Med Inform.

[42] Harari GM, Müller SR, Mishra V, Wang R, Campbell AT, Rentfrow PJ, Gosling SD (2017). An evaluation of students’ interest in and compliance with self-tracking methods: recommendations for incentives based on three smartphone sensing studies. Social Psychol Personality Sci.

[43] Mekhael M, Noujaim C, Lim CH, El Hajjar AH, Chaudhry HA, Lanier B, Chouman N, Makan N, Dagher L, Zhang Y (2022). COMPLIANCE CHALLENGES IN A LONGITUDINAL COVID-19 COHORT USING WEARABLES FOR CONTINUOUS MONITORING. Cardiovasc Digit Health J.

[44] Wang W, Harari GM, Wang R, Müller SR, Mirjafari S, Masaba K, Campbell AT (2018). Sensing behavioral change over time: using within-person variability features from mobile sensing to predict personality traits. Proc ACM Interact Mob Wearable Ubiquitous Technol.

[45] Hermsen S, Moons J, Kerkhof P, Wiekens C, De Groot M (2017). Determinants for sustained use of an activity Tracker: Observational Study. JMIR Mhealth Uhealth.

[46] Martinez GJ, Mattingly SM, Robles-Granda P, Saha K, Sirigiri A, Young J, Chawla N, De Choudhury M, D’Mello S, Mark G (2021). Predicting Participant Compliance with Fitness Tracker wearing and ecological momentary Assessment Protocols in Information workers: Observational Study. JMIR Mhealth Uhealth.

