# Supplementary material for: The Burnout PRedictiOn Using Wearable aNd ArtIficial IntelligEnce (BROWNIE) study: a decentralized digital health protocol to predict burnout in registered nurses
Source: BMC Nurs. 2024 Feb 13;23:114. doi: 10.1186/s12912-024-01711-8 (PMC10863108; doi:10.1186/s12912-024-01711-8)
Supplement: Supplementary file 1 — Additional file 1. [file 12912_2024_1711_MOESM1_ESM.docx]

**Supplementary Materials**

**S1. RATING SCALES AND INSTRUMENTS**

1. Maslach Burnout Inventory (MBI):  The MBI is a commonly used validated measure of burnout. The specific version of the MBI used in this study will be the MBI-Human Services survey (MBI-HSS), which is the original and most widely used version of the MBI, intended and validated for use for respondents working in a wide variety of occupations, including nurses, physicians, health aides, social workers, health counselors, therapists, police and correctional officers, clergy and others. The MBI-HSS has three scales: Emotional Exhaustion (EE, which measures feelings of being emotionally overextended and exhausted by one’s work); Depersonalization (DP, which assesses unfeeling and impersonal responses toward care recipients); and Personal Accomplishment (PA, which measures feelings of competence and successful achievement in one’s work). The MBI-HSS is a 22-item survey, with response items based on frequency, including never, a few times a year or less, once a month or less, a few times a month, once a week, a few times a week, or every day. Although the MBI-HSS was intended to be used to examine relationships with subscale scores as continuous measures, many investigators dichotomize results to define burnout being present when EE total score is 27+ or DP total score is 10+ (Dyrbye et al., 2009). These definitions can distinguish clinical burnout from non-burned out and defines a level of burnout that places individuals at higher risk of potentially serious personal and professional consequences (Dyrbye et al., 2008, 2010; Schaufeli et al., 2001; Shanafelt et al., 2010, 2011; West et al., 2006, 2009).
2. The Center for Epidemiologic Studies (CES-D):  The Center for Epidemiologic Studies Depression (CES-D) scale is one of the most frequently used self-report measures of depressive symptoms and experiences (Radloff 1977; Santor et al., 2006). The score is the sum of all 20 questions (range 0-60), with a score of 16 or higher defining that a respondent is depressed (sensitivity = 0.87, specificity = -.77) (Shean & Baldwin, 2008), using a scoring scheme that takes note of reverse scoring for questions 4, 8, 12, and 16. The CES-D has excellent reliability (Cronback’s alpha = 0.85-0.90; test-retest reliability = 0.45-0.54) and validity (based on moderate correlation with the HAMD, BDI-II, and Raskin Rating scale, 0.44-0.54) (Hann et al., 1999; Radloff 1977; Roberts 1980; Shean & Baldwin, 2008). More recently, the CES-D was shown to have convergent validity with the PHQ-9 (r = 0.85) (Amtmann et al., 2014). One particular advantage of the CES-D for the purposes of the current study is its mixture of negatively-worded items (e.g., “I felt sad,” etc.) and positively worded items (e.g., “I felt happy,” etc.), raising the possibility that the CES-D can be conceptualized as a measure for the depression/well-being continuum (Joseph 2007). Indeed, the CES-D has been shown to measure a continuum ranging from well-being to depression, with convergent validity with validated measures of well-being (Siddaway et al., 2017; Wood et al., 2010). In addition, the CES-D measures map to 8 subscales: sadness/dysphoria (items 2, 4, and 6); loss of interest/anhedonia (items 8 and 10); appetite (items 1 and 18); sleep (items 5, 11, and 19); thinking/concentration (items 3 and 20); guilt/worthlessness (items 9 and 17); tiredness/fatigue (items 7 and 16); movement/agitation (items 12 and 13); and isolation (items 14 and 15).
3. Linear Analog Self-Assessment (LASA): The LASA is a subject-rated quality of life (QOL) measure, the items for which have been validated as general measures of global QOL and dimension-specific QOL across numerous settings (Grunberg et al., 1996; Gudex et al., 1996; Hyland et al., 1996; Locke et al., 2007; Sriwatanakul et al., 1983; Wewers & Lowe 1990). Of relevance to this work, the LASA has been validated with 9295 individuals (Singh et al. 2014) and has been used in numerous clinical QOL trials and in healthy populations (Clark et al. 2013). In addition to measuring overall QOL, the LASA includes items that assess mental (intellectual), emotional, physical, spiritual, and social QOL. Higher overall QOL is defined as having an overall score of the median value or higher; others may be classified as having lower QOL (Campolong et al. 2016).
4. Multifactor Screener Food Intake Questionnaire. Multifactor screener assesses approximate intakes of fruits and vegetables, percentage energy from fat, and fiber. The screener asks respondents to report how frequently they consume foods in 16 categories. The screener also asks one question about the type of milk consumed. No portion size questions are asked. This screener does not attempt to assess total diet.
5. Occupational Exposure Survey (OES). The OES is an investigator-developed survey that assesses the frequency of adverse workplace exposures. The specific exposures surveyed are those listed in Table 1b (white boxes). Respondents will be asked to estimate whether they encountered the specified exposures.
6. Belonging Measure. The Belonging measure is a two-item validated tool which will be used to assess participants’ sense of belonging to their nursing unit and to their organization.
7. Human Flourishing Index (HFI). The HFI is a 12-item validated instrument that assesses flourishing across 6 life domains (happiness/life satisfaction, mental/physical health, meaning/purpose, character/virtue, close social relationships, financial/material stability; 2 items per domain; Weziak-Bialowolska, 2019; Vanderweele, 2017). This measure is copyrighted under a Creative Commons License (CC-BY-NC 4.0) and can be used without permission for non-commercial purposes if proper citation is given (Vanderweele, 2017).

**S2. TURNOVER QUESTIONAIRE**

**Factors Contributing to Change**

Thank you for participating in the BROWNIE study. We understand that your work environment/role is changing. We would like to better understand the reasons for the change. Please select a response to each possible factor contributing to your decision ranging from

**1 = Not Very to 5 = Very much**

1. Date
2. Seeking higher paid position*

1

2

3

4

5

1. Family or other life demands*

1

2

3

4

5

1. Dissatisfaction with work unit*

1

2

3

4

5

1. Retirement*

1

2

3

4

5

1. Seeking further education*

1

2

3

4

5

1. Dissatisfaction with organization*

1

2

3

4

5

1. Dissatisfaction with nursing career

1

2

3

4

5

1. Other reason (s)

1

2

3

4

5

If other, please state the reasons(s)

Your answer
